# Medical Officers of the Navy

**Published:** 1843-05-13

**Authors:** 


THE MEDICAL EXAMINER.

PHILADELPHIA, MAY 13, 1843.

### MEDICAL OFFICERS OF THE NAVY.

To appreciate how lowly medical service is estimated we must look at the duties performed by other officers and the salaries they receive, as well as the relative position assigned them by the regulations and usage of the navy. If we were to carry the comparison into civil life, perhaps the contrast would be equally striking.

Let us compare the career of a medical officer with that of a purser in the navy.

The duties performed by pursers require for their efficient discharge men who have a fair counting-house education, as far as the art and mystery of book-keeping is concerned, and a general knowledge of trade, that is, of buying and selling goods advantageously. The system of book-keeping adopted in the navy is of the simplest kind, a knowledge of which may be acquired in two or three weeks; and, as a general rule, the whole duty falls upon the purser's steward, now called purser's clerk; in fact, the affairs of his office require rather a general superintendence than attention to details. Such was the case, even when his salary was made up by a per cent. profit on goods of various kinds, sold to the men on board ship for their use, when it might be supposed personal interest would prompt a strict personal attention to affairs made up of many details. But since they receive an annual pay, it is probable they will be better able to delegate more of the duty to the clerk, for which, of course, the purser himself will be responsible to the government. This responsibility is guarantied by two or more securities to the amount of $25,000. As the expenditure of all monies for a ship, the pay of the officers and crew, provisions and clothing of the men, (the issue of rations,) falls within their province, the pecuniary responsibilities of pursers are very great; hundreds of thousands passing through their hands in the course of a year. The corps of pursers is a highly respectable body of gentlemen; instances of defalcation are almost unknown amongst them. But, notwithstanding their responsibilities and respectability, their relative position in the service is not very much better than that of surgeons, though it is far superior to that of all other medical officers.

By the law of August, 1842, pursers receive a yearly compensation as follows:

| | |
|---|---|
| Pursers under 5 years standing when on leave of absence, | $1000 |
| " over 5 and under 10 years standing, | 1200 |
| " over 10 and under 15 " " | 1400 |
| " over 15 and under 20 " " | 1600 |
| " over 20 years, | 1800 |
| When on duty, on a ship-of-the-line at sea, | 3573 |
| " " on a frigate or razee at sea, | 3073 |
| " " on a sloop of war and first class steamer, | 2073 |
| " " on a brig or schooner, or second class steamer, | 1573 |
| " " at the navy yard Boston, New York, Norfolk or Pensacola, | 2500 |
| " " at the navy yard Portsmouth, Philadelphia and Washington, | 2000 |
| " " at other naval stations in the United States, | 1500 |
| " " on receiving ships at Boston, New York and Norfolk, | 2500 |
| " " on receiving ships at other places, | 1500 |

This compensation is probably not more than enough to reward them for the $25,000 bonds, and their professional services. Although there is no law to prevent the youngest purser in the navy from being assigned to a ship-of-the-line, it is not likely to occur, because there is a sort of tacit understanding that they must render service progressively from the small to the large vessels. Perhaps it might be safe to say that pursers, for the first five years, serve in brigs and schooners; for the second, in sloops of war; for the third, in frigates; and after that in ships-of-the line; so that a purser will rarely be ordered to a line-of-battle-ship until after he has been fifteen years in the navy. But after ten years they may be assigned probably to either of the larger receiving vessels or navy yards.

Let us suppose two individuals, say at the age of twenty-five, to enter the navy on the same day; one leaving a book-keeper's situation in a counting house or store, with a purser's commission; the other quitting the place of resident physician in a hospital, or even fresh from the University, to serve as an assistant surgeon. Let us compare the position they occupy in the service, and the compensation received by them, and we may find how much medical education is undervalued by the government, or over estimated by the profession.

The purser enters upon his career with an annual pay of $1000, while unemployed; but the assistant surgeon, under the same circumstances, receives $650. Both may be, perchance, assigned for duty on board of the same sloop of war. The purser takes possession of one of the two best state-rooms in the ward-room, with an annual pay of $2073, and enjoys the same comforts, personal consideration, naval etiquette, and social position, as the lieutenants of the vessel; and in point of lodgement is better off than the surgeon. But the assistant surgeon, with his collegiate honours and his professional qualifications, stamped by the highest test in the country, is allowed a place to hang up his hammock or cot in the steerage, and is assigned to the companionship of midshipmen, boys of from fourteen years and upwards, and his annual compensation is $1023. While the purser departs from and enters the ship from the starboard side, with the honours of the side, the assistant surgeon passes in and out of the vessel by the larboard, with the midshipmen, stewards and servants, "unnoticed and unsung." And according to the received rules of etiquette, he ought not to pass the purser without the salute of touching his hat, because his position in the vessel is a subordinate one to all those who inhabit the ward-room. When both are on the quarter deck, the purser walks on the starboard side, but if the assistant surgeon goes there he is liable to be told he is trespassing.

Before a physician can receive a surgeon's commission he will have served, on an average, ten years in the navy, as assistant and passed assistant surgeon. Therefore, while he receives $1000 a year on leave, the purser, who entered the navy at the same time with him, will receive $1600 on leave; and while the surgeon, during the same period at sea, receives $1406, the purser will receive $3073; on shore duty the surgeon will receive $1250, but the purser will receive either $2000 or $2500, at one of the navy yards or receiving ships. Therefore, throwing out of the calculation all the disagreeable service which he performs as an assistant surgeon for ten years of his life, in a steerage or cockpit, while the purser has enjoyed the comforts of one of the best sleeping berths in the ward-room, in a pecuniary point of view, at the end of his ten years the medical officer finds his commission to be worth about one-half less than that of the purser who entered the navy on the same day.

Again: if we follow the medical officer and purser in their respective careers, at the end of twenty years from the day on which we have supposed both to enter the navy, we shall find that the purser, on leave, receives $1800, on shore duty $2500, and at sea $3573; or, in other words, the highest pay of his grade; but the surgeon, after the same period in the service, receives, on leave, $1400, on shore duty $1750, at sea $1939, or if, by any chance, he be fleet surgeon, $2173,—in other words, before the surgeon can receive the highest pay of his grade, that is, $1800 on leave, $2250 on shore, and $2773 at sea as fleet surgeon, he must have been thirty years in the navy, and have reached an age not usually calculated to encounter the discomforts of a sea-life in a ward-room. But the purser receives the highest pay of his grade after twenty years service.

It would be, perhaps, invidious to compare the mental qualifications, education, and professional acquirements requisite for the two grades; and it may be sufficient merely to direct attention to this point, to convince any thinking man that the educational qualifications of the surgeon are necessarily of an incomparably higher order than those of the purser. As to their comparative responsibility in a pecuniary point of view only, that of the purser is more extensive; though, as far as risk of loss is concerned, it is not more than that of the surgeon, who must account for a multitude of perishable and very destructible articles, often amounting to several thousands of dollars in value, for the preservation of which his means are usually very limited. The purser, on the contrary, is in every way assisted and facilitated (and most justly) in guarding against loss in his department, besides being allowed a fair per cent. discount for waste or loss in measuring, weighing, &c. But the surgeon's stores are very apt to be looked upon as a sort of necessary nuisance; and when a store-room is assigned for his use, it is sometimes looked upon rather in the light of a personal favour to himself than as a right. Even in frigates, surgeons have been obliged to stow a part of the medical outfit in their sleeping berth or state-room; and it has never been supposed there can be either waste or loss by weighing or measuring the articles in his keeping.

The following table fairly contrasts the compensation of pursers and medical officers, and will show, at a glance, the comparative money value of the two commissions, both present and prospective, through twenty years.

| | On leave. | Shore duty. | At sea. |
|---|---|---|---|
| Purser, first 5 years, | $1000 | $1500 | $1573 |
| Medical officer, first 5 years, (assistant surgeon,) | 650 | 950 | 1023 |
| Purser, second 5 years, | 1200 | 2000 | 2073 |
| Medical officer, second 5 years, (passed assistant surgeon,) | 850 | 1150 | 1273 |
| Purser, third 5 years, | 1400 | 2500 | 3073 |
| Medical officer, third 5 years, (surgeon,) | 1000 | 1250 | 1406 |
| Purser, fourth 5 years, | 1600 | 2500 | 3573 |
| Medical officer, fourth 5 years, (surgeon,) | 1200 | 1500 | 1673 |

Theology is not much better appreciated than medicine in the navy; but one had better serve ten years as chaplain than as assistant surgeon. When unemployed, chaplains receive yearly $800; when on duty on shore $1200, and at sea $1273—their pay not being progressive with years of service.

Professors of mathematics, in point of emolument, are on the same level with chaplains; both grades mess in the ward-room. The professors are usually occupied from two to four hours a-day on board ship, teaching those lower branches of mathematics which are essential to practical navigation; consequently, their professional qualifications are not, necessarily, of the highest order.

Secretaries mess in the ward-room, and receive $1073; their duties require them to write a good hand, and possess a fair English education.

On board of steamers the chief engineer messes in the ward-room, and receives annually $1575; and when on leave, $1200.

Whether medical officers are comparatively better or worse paid than chaplains, professors, secretaries and steam engineers, the reader can now judge for himself.

We have next to compare the career of a medical officer with that of a sea-officer in the navy.

The sea-officer enters the service, say at fifteen years of age, and receives the whole of his professional education at the cost of the government, and is fully supported during the time. At the age of twenty-five, at the same period of life when assistant surgeons are appointed, he receives a lieutenant's commission, and takes his place in the ward-room. When on leave, his yearly pay is $1200; when on shore duty, $1500; and when at sea, $1573. On an average, after serving eighteen years as a lieutenant, he is promoted to be a commander, and takes possession of his own cabin, when his annual pay is, on leave, $1800; on shore duty, $2100; and at sea, $2573. In this grade he serves, on an average, eight years, when he becomes a captain, with an annual pay, on leave, of $2500; on shore duty, $3500; at sea, $3573; and if in command of a squadron, $4073. Therefore, twenty-six after the assistant surgeon enters the navy, the lieutenant, who was commissioned on the same day, reaches the dignity and pay of a captain at the age of fifty-one years. But the medical officer, at the same age, is still in the ward-room, and his pay is $2206, (or, if a fleet surgeon, $2473.)

If a parent had two sons of the same age, both destined to the naval service, one as a sea-officer and the other as a medical officer, he would be charged with the care and education of one until he was fifteen, while the other would be an expense to him till he was twenty-five. And when both were fifty years of age, the son who had cost his father least, would be receiving, on leave, $2500; on shore duty, $3500; and at sea, $3573; while he who laboured most, and whose profession had been at his father's expense, would receive, on leave, $1600; on shore duty, $2000; and at sea, $2206.

It only remains to contrast the pay of medical officers of the navy with those of the army.

| | *Navy.* | *Army.* |
|---|---|---|
| Chief of the Medical Bureau, | $2500 | $3042 |
| Surgeons of ten years standing, | from $1400 to $2173 | From $2519 to $2824 |
| Surgeons of less than 10 years, | from $1000 to $1573 | " $1794 to $2227 |
| Assistant surgeons, | from $650 to $1273 | " $1083 to $2439 |

Besides being better paid, the medical officers of the army hold a better position relatively to officers of the line, than medical officers of the navy. Although medical service in the army is more highly appreciated in the army than in the navy, it is very far, in our opinion, from being too highly paid or estimated. None but those who are of the profession can fully understand the long and arduous labour and expense required to make a competent practitioner; and perhaps few persons know the toil, mental anxiety, and, consequently, wear and tear of health and constitution, that the practitioner of medicine and surgery must encounter, whether engaged in civil or military practice. We verily believe, if all these matters were clearly understood, the number of aspirants for medical honours would be very sensibly diminished; particularly if they would take into consideration the fact, which is too generally overlooked, that to be a successful practitioner of medicine, a capital sufficient to maintain a man as a gentleman for at least ten years after graduation is necessary.

The remark of Professor Gibson is probably true, "that the education of members of the profession, if complete, will often consume as much capital as would afford them, without practice, a decent competency."—*Rambles in Europe in* 1839.

At a time when the profession, both in England and the United States, is making efforts to augment its worth, and increase and sustain its respectability, the great body of practitioners ought not to overlook the condition of those members of the profession who are employed in military service, for, by securing their interests they protect themselves. In short, the members of the profession at large, in all parts of the country, are called upon, in professional fellowship, to exercise their influence in behalf of those medical men who serve their country either in the army or navy. Is the profession of medicine to be viewed by the government and by the people as inferior to that of arms, to that of a seamen, to that of a paymaster, or of a purser, and consequently be degraded in position and emolument, while the great mass of practitioners look on, and acknowledge that the science of medicine and surgery is really worth less to the public than the arts of arms or traffic?

Is it not time to teach the world that properly educated members of our profession are equal, in point of worth and respectability, to the members of any other class or calling in the United States? Instruct the people that the term physician does not include homœopathists, thomsonians, hydrosudopathists, electro-magnatists, or any other genus or species of quacks and impostors. If we permit these ignoble and miscalled doctors, by bold assumption, to place themselves upon a level with the legitimate members of the profession in the undiscriminating eye of the public, we deserve the degradation that surely awaits us. But before we can expect success, we must find some means of destroying the quackery that is said to exist among "regularly bred physicians." And perhaps a powerful mode of effecting this purpose would be, to introduce the fashion of cash payments, either for the visit or at the conclusion of the case, which would have the effect of removing temptation to resort to irregular modes of advertising and puffing from needy practitioners. This time seems to be propitious for establishing such a fashion, because in all directions we meet the significant placard, "terms cash," even in places where business was formerly done on the credit system. To those members of the profession whose practice only yields a support, the changing from credit to cash for the wants of themselves and families must be extremely inconvenient, while they are obliged to render their services on credit. And this inconvenience may result in quackery or starvation.

If the cash system were in vogue, as a general rule, physician's bills or fees would be more cheerfully paid than they are now. The services of the medical adviser, rendered in February, are apt to be coldly remembered the following December; and although the patient in the mean time may have paid the highest fees to the most distinguished lawyer, given the highest price for furniture, or dry goods, or wines, or contributed largely to his church, paying high pew rent—in short, paying the highest prices for the means of supplying all his wants and satisfying his tastes, the physician is expected to deduct something from his account at the end of the year. Besides being more advantageous to the physician, to the great majority of his patients it would be more agreeable and convenient to pay his fee daily, or to liquidate his bill at the termination of the case.

But who is to bring about a change that seems to be fraught with so much benefit to the mass of the profession? Can it be done by colleges of physicians or medical societies? Or is it in the power of a few leaders to establish the custom, by setting an example that the less influential members of the profession might follow without risk of losing business?

